# Racial/Ethnic Differences in Associations of Non-cigarette Tobacco Product Use With Subsequent Initiation of Cigarettes in US Youths

**DOI:** 10.1093/ntr/ntaa170

**Published:** 2020-09-19

**Authors:** Andrew C Stokes, Anna E Wilson, Dielle J Lundberg, Wubin Xie, Kaitlyn M Berry, Jessica L Fetterman, Alyssa F Harlow, Yvette C Cozier, Jessica L Barrington-Trimis, Kymberle L Sterling, Emelia J Benjamin, Michael J Blaha, Naomi M Hamburg, Aruni Bhatnagar, Rose Marie Robertson

**Affiliations:** 1 American Heart Association Tobacco Regulation and Addiction Center, Dallas, TX; 2 Department of Global Health, Boston University School of Public Health, Boston, MA; 3 Department of Epidemiology, University of Minnesota, Minneapolis, MN; 4 Evans Department of Medicine and Whitaker Cardiovascular Institute, Boston University School of Medicine, Boston, MA; 5 Department of Epidemiology, Boston University School of Public Health, Boston, MA; 6 Department of Preventive Medicine, University of Southern California, Los Angeles, CA; 7 School of Public Health, University of Texas Health Sciences Center, Dallas, TX; 8 Department of Medicine, The Johns Hopkins University, Baltimore, MD; 9 Department of Medicine, University of Louisville, Louisville, KY

## Abstract

**Introduction:**

Understanding which non-cigarette tobacco products precede smoking in youth across different racial/ethnic groups can inform policies that consider tobacco-related health disparities.

**Methods:**

We used nationally representative, longitudinal data from the Population Assessment of Tobacco and Health Study waves 1–4. The sample was a dynamic cohort of cigarette-naïve youth aged 12–17 years. Mixed-effects models were used to assess non-cigarette product (e-cigarette, cigar product, or other product) use with cigarette use over 1-year intervals.

**Results:**

Of the 28 788 observations pooled across waves 1–4, respondents were 48.7% non-Hispanic white, 13.9% non-Hispanic black, and 23.1% Hispanic. Odds of cigarette initiation over 1-year follow-up were higher among youth with prior use of e-cigarettes (odds ratio [OR], 2.76; 95% confidence interval [CI], 2.21–3.45), cigars (OR, 2.00; 95% CI, 1.42–2.80), or other products (OR, 1.66; 95% CI, 1.28–2.14) compared to never users. At the population level, 20.6% of cigarette initiation was attributable to e-cigarette use among white youth and 21.6% among Hispanic youth, while only 3.5% of cigarette initiation was attributable to e-cigarette use among black youth. In contrast, 9.1% of cigarette initiation for black youth was attributable to cigar use compared to only 3.9% for both white and Hispanic youth.

**Conclusions:**

Prior use of e-cigarettes, cigars, and other non-cigarette products were all associated with subsequent cigarette initiation. However, white and Hispanic youth were more likely to initiate cigarettes through e-cigarette use (vs. cigar or other product use), while black youth were more likely to initiate cigarettes through cigar use (vs. e-cigarette or other product use).

**Implications:**

Our findings suggest that previous studies on effects of non-cigarette tobacco products may overlook the critical role of cigar products as a pathway into cigarette smoking among US youth, particularly black youth. While our data support the importance of e-cigarette use as a pathway into smoking, regulatory actions aimed at addressing youth e-cigarette use alone may contribute to disparities in black versus white tobacco use and further exacerbate inequities in tobacco-related disease. Thus, contemporary policy development and discourse about the effects of non-cigarette tobacco products on cigarette initiation should consider cigar and other non-cigarette products as well as e-cigarettes.

## Introduction

Electronic cigarette (e-cigarette) use has grown rapidly over the last decade in the United States.^1,2^ Among high school students, past-30-day use of e-cigarettes rose from 1.5% in 2011 to 27.5% in 2019, with the greatest increases between 2017 and 2019.^3,4^ Trends in e-cigarette use initiation have alarmed public health officials concerned that e-cigarette use may renormalize tobacco use among youth,^5,6^ reversing decades of progress towards decreasing tobacco-related disease burden and mortality in the United States.^7^ In fact, a growing body of evidence suggests that e-cigarette use increases the risk of subsequent combustible cigarette smoking among youth and young adults by 3 to 4 times.^8–11^ At the population level, a recent study estimated that 43 000 current youth smokers and 178 000 ever youth smokers may have started smoking cigarettes as a result of e-cigarette use between 2013–2014 and 2015–2016.^8^

E-cigarette use is most common among white youth; in 2018, 32.4% of white high school-aged youth reported current e-cigarette use compared to 23.2% of Hispanic and 17.7% of black youth.^4^ Moreover, the association of e-cigarette use with subsequent cigarette initiation is stronger for white youth and young adults than for Hispanic youth and young adults.^12^ While rising e-cigarette use has captivated the attention of researchers, policy-makers, and media nationwide, the use of other non-cigarette tobacco products such as traditional cigars, little cigars, and cigarillos^13,14^ have received less attention. This is notable as cigar use has previously been associated with subsequent initiation of cigarette use.^15^ In 2018, 12.3% of black youth reported current cigar product use compared to 7.6% of white and 6.2% of Hispanic youth.^4^ Although e-cigarette use represents a potentially concerning pathway to cigarette use, especially among white youth, the use of other non-cigarette products may also pose a significant public health risk, especially for black youth and young adults.

Few studies have examined the association of both e-cigarettes and other non-cigarette tobacco products with subsequent cigarette initiation among youth. Even fewer have considered the important racial/ethnic differences that may exist.^16,17^ Thus, key stakeholders may overlook the role of various non-cigarette products apart from e-cigarettes as pathways into cigarette smoking. New longitudinal, nationally representative evidence is needed to understand how individual and population-level effects of e-cigarettes, cigars, and other non-cigarette tobacco products influence combustible cigarette initiation among youth, and whether the pathways to cigarette initiation differ by race/ethnicity. Such research will be particularly relevant to policy-makers to ensure that regulatory activities do not further exacerbate existing racial/ethnic inequities in tobacco-related disease, including cardiopulmonary disease.^18–22^

In this investigation, we use data from the Population Assessment of Tobacco and Health (PATH) Study to research associations of e-cigarette, cigar, and other non-cigarette product use with subsequent cigarette use among US youth overall and by race/ethnicity. Specifically, we test the hypotheses that (1) cigars and other non-cigarette products, in addition to e-cigarettes, are associated with progression to cigarette use, and (2) cigar use is a more important pathway into smoking than e-cigarette use among black youth.

## Methods

### Study Design and Sample

The PATH Study is a large, nationally representative longitudinal survey of tobacco use, attitudes, and history among youth and adults in the United States.^23,24^ The study uses a four-stage, stratified, probability sample design. We used four waves of PATH data from 2013 to 2018, with waves separated by 1 year. Data for Wave 1 were collected between 2013 and 2014, for Wave 2 between 2014 and 2015, for Wave 3 between 2015 and 2016, and for Wave 4 between December 2016 and January 2018.^23^

PATH replenishment samples were incorporated into our analysis at Waves 2 and 3,^24^ and participants who aged out of the youth survey (ages 18+) were censored from the remainder of the analysis. Because not all youth participated in each wave, this is considered a dynamic cohort. In order to capture all eligible data across the four waves, we stratified the waves into 3 intervals (Wave 1-Wave 2, Wave 2-Wave 3, and Wave 3-Wave 4). The first wave of each interval was defined as an *exposure wave* and the second as an *outcome wave*. Pooled across waves 1–4, respondents could contribute up to three interval observations to the analyses, or *person-intervals*. The sample was restricted to youth with data on race/ethnicity who were aged 12–17 years and cigarette naïve at exposure wave, with cigarette use status available for the wave immediately following the exposure wave (outcome wave). For example, if a participant was cigarette naïve at Wave 1, we would assess cigarette use status at Wave 2. Participants continued to be eligible if they were cigarette naïve at the beginning of each interval (ie, youth were censored after smoking initiation). Youth with missing outcome status (ever or past-30-day cigarette use) at any wave were excluded for a final analytic sample of 13 934 youth contributing 28 788 person-intervals (Supplementary eFigure 1).

Our analysis relied on de-identified data and was therefore exempted from review by the Boston University Medical Center Institutional Review Board. We followed the Strengthening the Reporting of Observational Studies in Epidemiology (STROBE) reporting guideline for observational studies.

### Non-cigarette Tobacco Product Use

Ever use of non-cigarette tobacco products (e-cigarette, cigar [traditional cigar, filtered cigar, cigarillo], and other product [hookah, smokeless tobacco, pipe, dissolvable tobacco, bidis, kreteks]) was defined as having ever tried the non-cigarette product, even one or two puffs, regardless of having used other non-cigarette products.

### Cigarette Use

Cigarette use at outcome wave was recorded in two ways: (1) if youth reported they had ever used a cigarette, even 1 or 2 puffs (cigarette initiation), or (2) if youth reported any use of a cigarette within 30 days prior to outcome wave (past-30-day use).

### Other Measures

Time-invariant covariates included sex, race/ethnicity (non-Hispanic white, non-Hispanic black, Hispanic, or non-Hispanic other), and parental education (< college degree, ≥ college degree). Time-variant covariates included age, living with a tobacco user (yes, no), and frequency of noticing warnings on cigarette packaging (never, rarely, sometimes, often, very often).

As in prior studies,^8^ we included six time-variant risk-taking indicators as covariates. These included (1) ever use of alcohol, (2) ever use of marijuana, (3) abuse of prescription drugs (Ritalin, Adderall, painkillers, sedatives, and tranquilizers), (4) curiosity toward cigarettes, (5) plans to smoke in the next year, and (6) openness to smoking if offered a cigarette by a friend. Youth were considered susceptible to cigarette use (yes/no) if one or more of questions 4–6 were answered “yes.” Time-invariant covariates were measured at the first observation for each respondent, and time-variant covariates were measured at each exposure wave.

### Statistical Analysis

Multilevel mixed-effects models for repeated measures were used to examine the association of non-cigarette tobacco product use (e-cigarette, cigar, and other product use) with cigarette initiation and past-30-day cigarette use. Multilevel modeling accounts for non-independence of observations. Models were run using the overall sample, and then for each racial/ethnic group (non-Hispanic white, non-Hispanic black, Hispanic). Due to insufficient sample size, non-Hispanic other youth were not included in analyses. We fit models with random effects at level-2 (person-level) and adjusted for all covariates in addition to non-cigarette tobacco products as risk indicators for subsequent cigarette use.

To account for missing data in covariates and exposures, we used multiple imputation by chained equations (7 imputations).^25^ To check the robustness of our models to bias from missing data, we constructed our regression models prior to imputation using the complete case sample and compared the estimates.

Finally, to estimate the fraction of incident cigarette use that could be explained by preceding non-cigarette tobacco use, we calculated population attributable fractions (PAFs) using the method described by Mansournia and Altman, in which the observed number of cases and expected number of cases under no exposure were derived from marginal standardization using risk estimates from regression models.^26^ Since our outcomes were rare (4.1% cigarette initiation, 2.0% past-30-day), our odds ratios approximate risk ratios. We used PATH sample weights to extend these estimates to the population-level, estimating the number of new youth cigarette smokers in the US attributable to each initial tobacco product type. This process was repeated using the stratified models to establish population-level estimates for each product class by race/ethnicity.

Data were analyzed using Stata, version 15 (StataCorp). We applied PATH derived sample weights from the most recently available observation for each respondent to adjust for unequal probabilities of selection and non-response.^24^ Variances were estimated using Taylor series linearization with the survey routine and tested statistical significance using a 2-sided test, with a significance level of .05.

## Results

The sample included 28 788 person-intervals constituted by 13 934 unique person-level observations. Person-intervals were 48.7% female, 53.3% non-Hispanic white, 13.9% non-Hispanic black, and 23.1% Hispanic, with mean age of 14.3 years. Additionally, 10.8% of exposure wave observations had ever tried ≥1 non-cigarette products and 2.3% had used a non-cigarette product within the past 30 days. Within the total sample, 6.3% of participants had ever tried e-cigarettes, 1.9% had ever tried cigars, and 4.9% had ever tried other tobacco products. Among those who had ever tried other tobacco products, 61.9% had tried hookah, and 33.7% had tried smokeless tobacco. Across outcome waves, 4.1% of observations involved ever cigarette use and 2.0% involved past-30-day cigarette use (Table 1).

**Table 1.** Characteristics of Person-Interval Observations, Population Assessment of Tobacco and Health Study, 2013–2018^a^

	Overall (*n* = 29 788)	NH white (*n* = 14 025)	NH black (*n* = 4187)	Hispanic (*n* = 8824)
	%^b^	%^b^	%^b^	%^b^
Exposure wave^c^				
Female	48.7	48.1	50.2	48.8
Age, y				
12	18.6	18.3	16.6	19.8
13	18.4	18.2	17.9	19.0
14	17.8	17.9	18.1	17.7
15	16.9	17.2	17.1	16.6
16	15.3	15.5	16.3	14.6
17	12.9	12.9	13.9	12.4
Parent completed college or higher	40.1	50.3	28.2	17.8
Lives with tobacco user	31.6	33.9	36.7	25.2
Frequency of noticing tobacco warnings				
Never	55.5	56.8	54.3	53.3
Rarely	16.6	16.5	15.8	17.7
Sometimes	11.9	12.0	11.3	12.3
Often	8.6	8.3	8.6	8.9
Very often	7.4	6.5	10.0	7.8
Ever used alcohol	26.1	30.1	19.6	21.4
Ever used marijuana	2.4	2.1	2.1	3.5
Ever abused prescription drugs	6.6	5.9	8.7	6.7
Plans to smoke in next year^d^	13.3	12.6	12.1	16.4
Ever been curious about cigarettes^d^	22.2	22.0	20.5	22.8
Would smoke if offered cigarette by friend^d^	15.3	15.2	12.7	17.8
Ever use^e^				
E-cigarette	6.3	6.5	5.2	6.8
Cigar	1.9	1.9	3.6	1.1
Other non-cigarette tobacco product	4.9	4.8	4.6	5.9
Past-30-day use				
E-cigarette	1.2	1.4	1.0	1.0
Cigar	0.4	0.4	0.9	0.2
Other non-cigarette tobacco product	0.9	0.9	0.8	0.9
Single use (only ever one product)				
E-cigarette	4.1	4.1	3.1	4.7
Cigar	0.7	0.6	2.1	0.5
Other non-cigarette tobacco product	2.7	2.5	2.5	3.6
Poly use (>1 product types)	2.2	2.5	2.1	1.9
Outcome wave				
Cigarette use initiation				
Cigarette ever	4.1	4.7	2.6	4.0
Cigarette current	2.0	2.3	1.3	1.8

e-cigarette, electronic cigarette; NH, non-Hispanic.

^a^Person-interval count. Intervals included respondents with data for at least two consecutive exposure wave and outcome waves, creating the potential for up to three time-varying within-person observations per respondent (W1-W2, W2-W3, W3-W4).

^b^Percentages were weighted using the most recent sample weight available per person. Guidelines for the Restricted-Use Files of the Population Assessment of Tobacco and Health Study prohibit the reporting of cell counts.

^c^Exposure wave is defined as the first wave within each interval. Follow-up was defined as the last wave within each interval, 1-year after baseline.

^d^For cigarette-susceptibility questions, responses of not at all or definitely not were considered nonsusceptible. All other responses were considered susceptible.

^e^Youths were considered to have prior non-cigarette tobacco use if they started using e-cigarettes, cigars, or other non-cigarette tobacco products prior to the interval exposure wave.

In the total sample, ever e-cigarette use (odds ratio [OR], 2.76; 95% confidence interval [CI], 2.21–3.45), cigar use (OR, 2.00; 95% CI, 1.42–2.80), and other product use (OR, 1.66; 95% CI, 1.28–2.14) were all significantly associated with increased odds of cigarette initiation over 1-year of follow-up compared with never users (Table 2). Additionally, the odds of past-30-day use were higher among youth with prior e-cigarette use (OR, 2.72; 95% CI, 2.00–3.68), cigar use (OR, 1.91; 95% CI, 1.19–3.07), and other product use (OR, 1.83; 95% CI, 1.26–2.65) compared with never users.

**Table 2.** Association of Non-cigarette Tobacco Product Ever Use With Subsequent Cigarette Use in Overall Sample, Population Assessment of Tobacco, and Health Study, 2013–2018 (*n* = 29 788)^a^

	Cigarette ever use		Cigarette past-30-day use	
Use at exposure wave ^b^	Weighted, unadjusted cigarette ever use, %^c^	OR (95% CI)^d^	Weighted, unadjusted cigarette past 30-d use, %^c^	OR (95% CI)^d^
E-cigarette				
Never	3.2	1 [Reference]	1.5	1 [Reference]
Ever	17.2	2.76 (2.21–3.45)	8.8	2.72 (2.00–3.68)
Cigar				
Never	3.8	1 [Reference]	1.8	1 [Reference]
Ever	20.4	2.00 (1.42–2.80)	10.9	1.91 (1.19–3.07)
Other				
Never	3.5	1 [Reference]	1.6	1 [Reference]
Ever	15.2	1.66 (1.28–2.14)	8.1	1.83 (1.26–2.65)

e-cigarette, electronic cigarette; OR, odds ratio; CI, confidence interval.

^a^Person-interval count. Intervals included respondents with data for at least two consecutive exposure wave and outcome waves, creating the potential for up to three time-varying within-person observations per respondent (W1-W2, W2-W3, W3-W4).

^b^Exposure wave is defined as the first wave within each specific interval. Youths were considered to have prior non-cigarette tobacco use if they started using e-cigarettes, cigars, or other non-cigarette tobacco products prior to interval exposure wave.

^c^Percentages were weighted using the most recent sample weight available per person. Guidelines for the Restricted-Use Files of the Population Assessment of Tobacco and Health Study prohibit the reporting of cell counts.

^d^Regression models were adjusted for sex, age, race/ethnicity, parental education level (bachelors or higher), ever alcohol use, ever marijuana use, ever prescription drug abuse, interval, and cigarette susceptibility.

**Table 3.** Association of Non-cigarette Tobacco Product Ever Use with Subsequent Cigarette Use by Race, Population Assessment of Tobacco and Health Study, 2013–2018 (*n* = 29 788)^a^

	Cigarette ever use		Cigarette past-30-day use	
Use at exposure wave^b^	Weighted, unadjusted cigarette ever use, %^c^	OR (95% CI)^d^	Weighted, unadjusted cigarette past 30-d use, %^c^	OR (95% CI)^d^
White: (*n* = 14 025)				
E-cigarette				
Never	3.6	1 [Reference]	1.7	1 [Reference]
Ever	20.3	3.05 (2.19–4.24)	10.7	3.04 (1.98–4.66)
Cigar				
Never	4.4	1 [Reference]	2.1	1 [Reference]
Ever	23.6	1.76 (1.08–2.85)	11.4	1.37 (0.69–2.72)
Other				
Never	4.1	1 [Reference]	1.9	1 [Reference]
Ever	18.2	1.66 (1.14–2.41)	9.7	1.75 (1.03–2.96)
Black (*n* = 4187)				
E-cigarette				
Never	2.3	1 [Reference]	1.1	1 [Reference]
Ever	7.6	1.35 (0.70–2.58)	4.3	1.18 (0.51–2.74)
Cigar				
Never	2.2	1 [Reference]	1.0	1 [Reference]
Ever	11.4	2.60 (1.36–4.98)	7.6	2.68 (1.21–5.93)
Other				
Never	2.4	1 [Reference]	1.2	1 [Reference]
Ever	6.0	1.12 (0.48–2.61)	3.7	1.37 (0.50–3.77)
Hispanic (*n* = 8824)				
E-cigarette				
Never	3.1	1 [Reference]	1.4	1 [Reference]
Ever	16.6	3.34 (2.24–4.99)	7.1	2.66 (1.44–4.92)
Cigar				
Never	3.8	1 [Reference]	1.6	1 [Reference]
Ever	22.2	2.42 (1.09–5.36)	12.2	3.22 (1.08–9.61)
Other				
Never	3.4	1 [Reference]	1.5	1 [Reference]
Ever	12.3	1.53 (0.97–2.40)	6.6	1.90 (0.96–3.76)

e-cigarette, electronic cigarette; OR, odds ratio; CI, confidence interval.

^a^Ns denote Person-interval counts. Intervals included respondents with data for at least two consecutive exposure wave and outcome waves, creating the potential for up to three time-varying within-person observations per respondent (W1-W2, W2-W3, W3-W4).

^b^Exposure wave is defined as the first wave within each specific interval. Youths were considered to have prior non-cigarette tobacco use if they started using e-cigarettes, cigars, or other non-cigarette tobacco products prior to the interval exposure wave.

^c^Percentages were weighted using the most recent sample weight available per person. Guidelines for the Restricted Use Files of the Population Assessment of Tobacco and Health Study prohibit the reporting of cell counts.

^d^Regression models were adjusted for sex, age, race/ethnicity, parental education level (bachelors or higher), ever alcohol use, ever marijuana use, ever prescription drug abuse, interval, and cigarette susceptibility

After stratifying by race/ethnicity, we found that non-Hispanic white youth (OR, 3.05; 95% CI, 2.19–4.24) and Hispanic youth (OR, 3.34; 95% CI, 2.24–4.99) with prior e-cigarette use had higher odds of cigarette initiation compared to never users. Youth in all three racial/ethnic groups with prior cigar use had higher odds of cigarette initiation compared to never users (non-Hispanic white youth [OR, 1.76; 95% CI, 1.08–2.85]; Hispanic youth [OR, 2.42; 95% CI, 1.09–5.36]; black youth [OR, 2.60; 95% CI, 1.36–4.98]). Only non-Hispanic white youth (OR, 1.66; 95% CI, 1.14–2.41) with ever other product use had higher odds of cigarette initiation compared to never users.

Similar associations were seen between prior non-cigarette tobacco products use with past-30-day cigarette use. Non-Hispanic white youth (OR, 3.04; 95% CI, 1.98–4.66) and Hispanic youth (OR, 2.66; 95% CI, 1.44–4.92) with ever e-cigarette use had higher odds of past-30-day cigarette use compared to never users; Hispanic youth (OR, 3.22; 95% CI, 1.08–9.61), and black youth (OR, 2.68; 95% CI, 1.21–5.93) with ever cigar use had higher odds of past-30-day cigarette use compared to never users; and non-Hispanic white youth (OR, 1.75; 95% CI, 1.03–2.96) with ever other product use had higher odds of past-30-day cigarette use compared to never users. Sensitivity analyses restricted to youth without missing data produced similar results for models run with overall sample and each racial/ethnic group (Supplementary eTable 1 and eTable 2).

**Figure 1.**
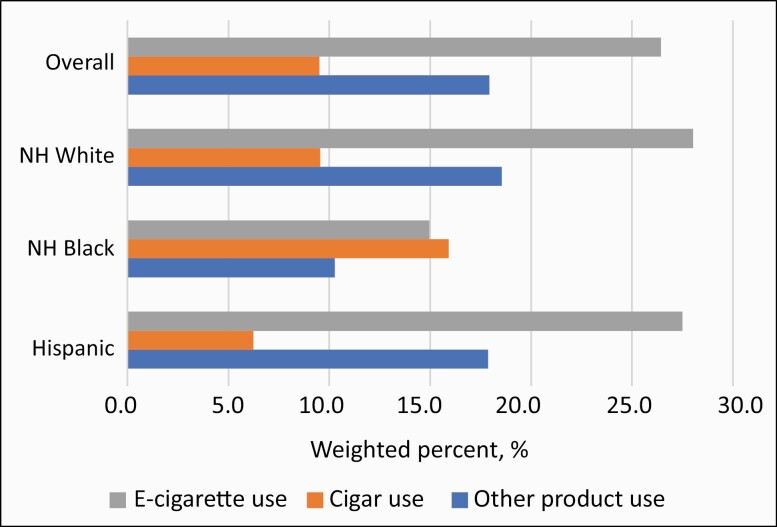
Prevalence of prior non-cigarette tobacco product use among youth with cigarette ever use, Population Assessment of Tobacco and Health Study, 2013–2018 (*n* = 1267)^a,b^. Abbreviations: e-cigarette, electronic cigarette. ^a^Percentages were weighted using the most recent PATH sample weight available per person. Guidelines for the Restricted Use Files of the Population Assessment of Tobacco and Health prohibit the reporting of cell counts. Results for non-Hispanic other youth are not included due to low sample size and lack of precision. ^b^Youths were considered to have prior non-cigarette tobacco use if they started using e-cigarettes, cigars, or other non-cigarette tobacco products prior to the interval baseline.

Figure 1 shows the distribution of non-cigarette products previously used among youth with cigarette initiation, by racial/ethnic group. Within this population, 28.1% of non-Hispanic white and 27.5% of Hispanic youth had ever used e-cigarettes compared to 15.0% of non-Hispanic black youth. For cigar products, the opposite pattern was observed: 15.9% of non-Hispanic black youth smokers had used cigars compared to 9.5% of non-Hispanic white youth and 6.2% of Hispanic youth.

At the population level, the fraction of cigarette initiation attributable to ever e-cigarette use was 17.9%; to cigar use, 4.6%; and to other product use, 6.9%. Estimates from our sample suggest that approximately 948 000 youth initiated cigarettes over the 1-year period (approximately 2017) during interval 3, the most recent interval. Approximately 170 000 cases of youth cigarette initiation can be attributed to prior e-cigarette use, 44 000 to prior cigar use, and 65 000 to prior other product use. Furthermore, the fraction of past-30-day use attributable to prior e-cigarette use was 22.6%; to cigar use, 6.8%; and to other product use, 11.8% (Table 4).

**Table 4.** Population-Level Proportion of Cigarette Use Attributable to Prior Use of Non-cigarette Tobacco Products, Population Assessment of Tobacco and Health Study^a^

	Cigarette ever use		Cigarette past-30-day use	
Prior use	PAF,^b^ %	Attributable users in 2017, No.^c,d,e^	PAF,^b^ %	Attributable users in 2017, No.^c,d,e^
Overall				
Ever e-cigarette use	17.9	170 000	22.6	108 000
Ever cigar use	4.6	44 000	6.8	33 000
Ever other product use	6.9	65 000	11.8	57 000
NH white				
Ever e-cigarette use	20.6	118 000	26.0	77 000
Ever cigar use	3.9	22 000	3.2	9000
Ever other product use	7.2	41 000	11.2	33 000
NH black				
Ever e-cigarette use	3.5	3000	2.3	1000
Ever cigar use	9.1	7000	12.2	5000
Ever other product use	1.0	1000	3.1	1000
Hispanic				
Ever e-cigarette use	21.6	50 000	22.1	26 000
Ever cigar use	3.9	9000	9.0	10 000
Ever other product use	6.1	14 000	13.3	15 000

e-cigarette, electronic cigarette; PAF, Population Attributable Fraction; CI, confidence interval.

^a^Results for non-Hispanic Other youth are not included due to low sample size and lack of precision.

^b^PAF = (observed number of cases - expected number of cases under no exposure)/observed number of cases, where both components were derived from marginal standardization using risk estimates from the prior regression models.

^c^New cigarette use was measured after 1-year follow-up at Wave 4. Data were collected for Wave 4 between December 2016 and January 2018. Results were rounded to the nearest 1000.

^d^Attributable Users = (PAF)*(Total New Users between Wave 3 and Wave 4).

^e^Total new ever cigarette users between Wave 3 and Wave 4 was 948 022 for the overall sample, 573 940 for non-Hispanic white, 77 354 for non-Hispanic black, and 232 437 for Hispanic youth. Total new past-30-days between Wave 3 and Wave 4 was 479 960 for the overall sample, 292 042 for non-Hispanic white, 40 203 for non-Hispanic black, and 115 767 for Hispanic youth.

Our stratified analysis indicated that 20.6% of cigarette initiation was attributable to e-cigarette use among white youth and 21.6% among Hispanic youth, while only 3.5% was attributable to e-cigarette use among black youth. Similarly, other product use was responsible for 7.2% and 6.1% of cigarette initiation among non-Hispanic white and Hispanic youth, respectively and responsible for only 1.0% of ever cigarette use among non-Hispanic black youth. In contrast, 9.1% of cigarette initiation among non-Hispanic black youth was attributable to cigar products compared to only 3.9% for both non-Hispanic white and Hispanic youth (Table 4).

## Discussion

In this nationally representative, longitudinal study of cigarette-naïve youth, prior use of e-cigarettes, cigar products, and other non-cigarette tobacco products were all significantly associated with increased likelihood of subsequent cigarette initiation in the overall sample. However, in stratified analyses we found racial/ethnic differences regarding which non-cigarette tobacco products were most strongly associated with youth cigarette initiation. Importantly, white and Hispanic youth were more likely to initiate cigarettes through e-cigarette use (vs. cigar use or other product use), while black youth were more likely to initiate cigarettes through cigar use (vs. e-cigarette or other product use). Evidence from this study suggests that in addition to e-cigarettes, multiple other non-cigarette tobacco products have the potential to place youth on a pathway to cigarette smoking, and that effects of specific non-cigarette products on cigarette initiation may vary by race/ethnicity. These findings should be explored in future analyses with access to more granular data on racial/ethnic differences in tobacco use behaviors.

Prior studies have shown that little cigars and cigarillo smokers are more likely to be young, black or Hispanic, with low socioeconomic status.^27,28^ Little cigars and cigarillos are often cheaper than other tobacco products due to lower taxation, and can be sold in smaller packages due to absence of pack size regulations.^17,29^ The availability of flavored little cigars and cigarillos and lower prices make them particularly appealing to youth smokers.^30,31^ Most young adults who use cigars purchase flavored products. Flavors mask the bitterness of tobacco and reduce the pain sensations and irritation caused by combustible tobacco product use.^15,31^ Additionally, tobacco advertisements and outlets are significantly more common in black, Hispanic, and low-income communities.^32,33^ This is notable given that both exposure to tobacco marketing and proximity to tobacco outlets are positively associated with tobacco initiation among youth and young adults.^34^

A significant body of research has documented the “Black smoking paradox”—the phenomenon that (1) despite social disadvantage and structural racism, black youth and young adults use cigarettes at lower rates than white youth, yet (2) tobacco-related diseases disproportionally affect black adults.^35,36^ Thus, although white youth use tobacco at higher rates, the burden of tobacco-related disease is distributed inequitably. While the tobacco paradox is not fully understood, prior studies have found evidence that exposure to racial discrimination, poverty, neighborhood segregation, comorbidities, structural barriers to health insurance and health care, lack of access to affordable cessation resources, and the use of menthol cigarettes could all be important drivers of the inequities observed in tobacco-related disease.^35,37–39^

While the results of this study should be interpreted with caution due to our limited sample size, the findings represent important hypothesis-generating evidence that strengthens the rational for future research to focus on racial/ethnic differences in non-cigarette tobacco product use. Additionally, if further confirmed, the evidence from this study has significant implications for the development of equitable, comprehensive tobacco regulations.

In 2009, the Family Smoking Prevention and Tobacco Control Act banned all flavors other than menthol from cigarettes,^40^ and in January 2020, the FDA issued a policy prioritizing enforcement against unauthorized flavored e-cigarette products popular among youth.^41^ Despite these efforts and the FDA’s regulatory authority over cigar products through the Deeming Rule,^42^ flavored cigar products that appeal to adolescents, including fruit and mint, remain on the market.^43^ According to our estimates, decreasing the cigar use among youth could prevent up to 4.6% of cigarette initiation overall and 9.1% among black youth specifically. In addition, this study suggests that FDA actions to regulate or ban e-cigarettes without taking concurrent actions on cigars may increase black versus white disparities in youth cigarette use.

Future efforts to reduce tobacco marketing to youth should consider limiting the sale of all flavored tobacco products to age-restricted, adult-only locations with increased age verification requirements,^44^ and increasing surveillance and regulation of tobacco advertisements and promotions on social media. Social media platforms have recently become major marketing arenas for tobacco products, and although nearly all youth in the United States use social media daily, few policies limit the extent to which tobacco brands can reach youth through these channels.^45^ Other possible regulatory actions include establishing buffer zones to prevent the sale of flavored tobacco products within 1000 feet of schools,^40,46^ and regulating cigar product taxation and package size.^47,48^ Additionally, ongoing investment is needed for community-level tobacco prevention and cessation programs that are culturally and contextually relevant to youth and young adults.

### Limitations

Our study had several limitations. First, since PATH data are observational, it was not possible to eliminate the possibility of residual confounding by measured and unmeasured factors or to establish causal relations. Second, we were unable to examine the effect of product characteristics or use behaviors such as dual/poly use on the likelihood of cigarette initiation by race/ethnicity due to insufficient sample size. Third, also due to limited sample size, we could not assess racial/ethnic differences for youth who were not white, black, or Hispanic. Future research with access to larger datasets should examine patterns of risk and tobacco use behaviors across additional racial/ethnic groups. Fourth, since non-cigarette product use was established at each exposure wave, our study may be subject to prevalent exposure bias and exposure misclassification by youths who began using non-cigarette tobacco products after baseline but before follow-up. Lastly, our PAF calculations are dependent on an assumption of causality between exposure and outcome, and should thus be considered as provisional until confirmed by further evidence.

## Conclusion

In this longitudinal study of cigarette-naïve youth, we found that multiple non-cigarette tobacco products are associated with cigarette initiation. However, our findings suggest that previous studies on effects of non-cigarette tobacco products may overlook the critical role of cigar products as a pathway into cigarette smoking among US youth, particularly black youth. While our data support the importance of e-cigarette use as a pathway into cigarette smoking, regulatory actions aimed at addressing youth e-cigarette use alone may contribute to disparities in black versus white tobacco use and further exacerbate inequities in tobacco-related disease. Thus, contemporary policy development and discourse about the effects of non-cigarette tobacco products on cigarette initiation should consider cigar and other non-cigarette products as well as e-cigarettes. These findings establish a strong scientific premise for future research to further investigate the role of race and ethnicity risk of youth cigarette initiation through non-cigarette tobacco products.

## Supplementary Material

A Contributorship Form detailing each author’s specific involvement with this content, as well as any supplementary data, are available online at https://academic.oup.com/ntr.

ntaa170_suppl_Supplementary_Materials

ntaa170_suppl_Supplementary_Taxonomy_Form
