# Supplementary material for: Racial/Ethnic Differences in Associations of Non-cigarette Tobacco Product Use With Subsequent Initiation of Cigarettes in US Youths
Source: Nicotine Tob Res. 2020 Sep 19;23(6):900–8. doi: 10.1093/ntr/ntaa170 (PMC8150136; doi:10.1093/ntr/ntaa170)

Racial/Ethnic Differences in Associations of Noncigarette Tobacco Product Use with Subsequent Initiation of Cigarettes in US Youths

Additional Results

eTable 1. Sensitivity to Missing Data: Association of Noncigarette Tobacco Product Use with Subsequent Cigarette Use, Population Assessment of Tobacco and Health Study, 2013-2018 (n=26,072)

eTable 2. Sensitivity to Missing Data: Association of Noncigarette Tobacco Product Use with Subsequent Cigarette Use by Race, Population Assessment of Tobacco and Health Study, 2013-2018 (n=26,027)

eFigure. Sample inclusion criteria

eTable1. Sensitivity to Missing Data: Association of Noncigarette Tobacco Product Ever Use with Subsequent Cigarette Use, Population Assessment of Tobacco and Health Study, 2013-2018 (n=26,072)[a]

Use at exposure wave [b]	Cigarette Ever Use		Cigarette Past-30-day Use	
	Weighted, unadjusted cigarette ever use, %[c]	OR (95% CI)[d]	Weighted, unadjusted cigarette past-30-d use, %[c]	OR (95% CI)[d]
**E-cigarette**				
**never**	3.2	1 [Reference]	1.5	1 [Reference]
**ever**	17.1	2.78 (2.17-3.56)	8.4	2.67 (1.91-3.73)
**Cigar**				
**never**	3.7	1 [Reference]	1.7	1 [Reference]
**ever**	23.5	2.26 (1.52-3.37)	12.0	1.97 (1.13-3.42)
**Other**				
**never**	3.5	1 [Reference]	1.6	1 [Reference]
**ever**	16.0	1.78 (1.34-2.37)	8.3	1.95 (1.91-2.99)

Abbreviation: e-cigarette, electronic cigarette; OR, odds ratio; CI, confidence interval
[a] Person-interval count. Intervals included respondents with data for baseline and follow-up, creating the potential for up to three time-varying within-person observations per respondent.
[b] Exposure wave is defined as the first wave within each specific interval. Youths were considered to have prior noncigarette tobacco use if they started using e-cigarettes, cigars, or other noncigarette tobacco products prior to interval baseline.
[c] Percentages were weighted using the most recent sample weight available per person. Guidelines for the Restricted Use Files of the Population Assessment of Tobacco and Health Study prohibit the reporting of cell counts.
[d] Regression models were adjusted for sex, age, race/ethnicity, parental education level (bachelors or higher), ever alcohol use, ever marijuana use, ever prescription drug abuse, interval, and cigarette susceptibility

eTable2. Sensitivity to Missing Data: Association of Noncigarette Tobacco Product Ever Use with Subsequent Cigarette Use by Race, Population Assessment of Tobacco and Health Study, 2013-2018 (n=26,027)[a]

	Cigarette Ever Use		Cigarette Past-30-day Use	
Use at exposure wave [b]	Weighted, unadjusted cigarette ever use, %[c]	OR (95% CI)[d]	Weighted, unadjusted cigarette past-30-d use, %[c]	OR (95% CI)[d]
**White: (n=12,591)**				
**E-cigarette**				
**never**	3.7	1 [Reference]	1.7	1 [Reference]
**ever**	20.2	2.80 (2.03-3.85)	10.5	2.77 (1.82-4.23)
**Cigar**				
**never**	4.4	1 [Reference]	2.1	1 [Reference]
**ever**	23.6	1.65 (1.00-2.74)	11.5	1.29 (0.63-2.61)
**Other**				
**never**	4.1	1 [Reference]	1.9	1 [Reference]
**ever**	18.6	1.65 (1.13-2.41)	9.9	1.76 (1.02-3.04)
**Black (n=3,483)**				
**E-cigarette**				
**never**	2.2	1 [Reference]	1.0	1 [Reference]
**ever**	7.6	1.44 (0.67-3.08)	4.3	1.36 (0.51-3.62)
**Cigar**				
**never**	2.2	1 [Reference]	1.0	1 [Reference]
**ever**	16.8	3.96 (1.84-8.50)	9.1	3.06 (1.18-7.94)
**Other**				
**never**	2.3	1 [Reference]	1.1	1 [Reference]
**ever**	6.6	1.67 (0.60-4.61)	3.6	2.18 (0.59-8.00)
**Hispanic (n= 7,629)**				
**E-cigarette**				
**never**	2.9	1 [Reference]	1.2	1 [Reference]
**ever**	15.8	4.08 (2.36-7.05)	5.5	2.72 (1.24-6.01)
**Cigar**				
**never**	3.5	1 [Reference]	1.4	1 [Reference]
**ever**	26.5	5.11 (1.76-14.90)	13.1	6.41 (1.56-26.39)
**Other**				
**never**	3.2	1 [Reference]	1.3	1 [Reference]
**ever**	12.1	1.93 (1.12-3.31)	5.6	2.36 (1.02-5.44)

Abbreviation: e-cigarette, electronic cigarette; OR, odds ratio; CI, confidence interval

[a] Person-interval count. Intervals included respondents with data for at least two consecutive exposure wave and outcome waves, creating the potential for up to three time-varying within-person observations per respondent (W1-W2, W2-W3, W3-W4).

[b] Exposure wave is defined as the first wave within each specific interval. Youths were considered to have prior noncigarette tobacco use if they started using e-cigarettes, cigars, or other noncigarette tobacco products prior to interval exposure wave.

[c] Percentages were weighted using the most recent sample weight available per person. Guidelines for the Restricted Use Files of the Population Assessment of Tobacco and Health Study prohibit the reporting of cell counts.

[d] Regression models were adjusted for sex, age, race/ethnicity, parental education level (bachelors or higher), ever alcohol use, ever marijuana use, ever prescription drug abuse, interval, and cigarette susceptibility

eFigure. Sample inclusion criteria

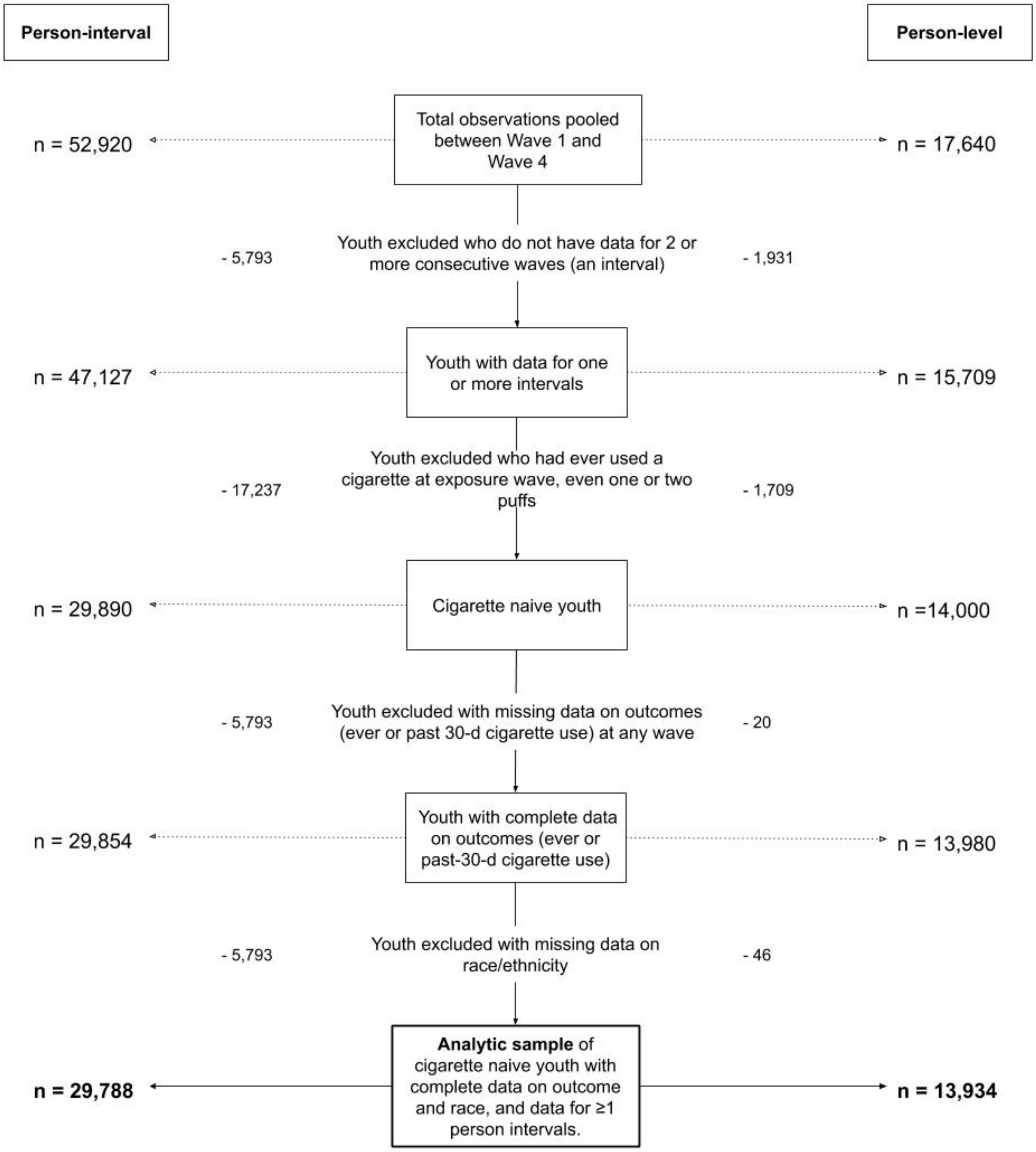

Supplement: ntaa170_suppl_Supplementary_Materials [file ntaa170_suppl_supplementary_materials.pdf]
