# Supplementary material for: Racial/Ethnic Differences in Associations of Non-cigarette Tobacco Product Use With Subsequent Initiation of Cigarettes in US Youths
Source: Nicotine Tob Res. 2020 Sep 19;23(6):900–8. doi: 10.1093/ntr/ntaa170 (PMC8150136; doi:10.1093/ntr/ntaa170)
Supplement: ntaa170_suppl_Supplementary_Taxonomy_Form [file ntaa170_suppl_supplementary_taxonomy_form.pdf]

CRediT Taxonomy – Stokes, CVD Biomarkers

Contributor Role	Role Definition
Conceptualization	Ideas; formulation or evolution of overarching research goals and aims
Methodology	Development or design of methodology; creation of models.
Software	Programming, software development; designing computer programs; implementation of the computer code and supporting algorithms; testing of existing code components.
Validation	Verification, whether as a part of the activity or separate, of the overall replication/reproducibility of results/experiments and other research outputs.
Formal Analysis	Application of statistical, mathematical, computational, or other formal techniques to analyze or synthesize study data.
Investigation	Conducting a research and investigation process, specifically performing the experiments, or data/evidence collection.
Resources	Provision of study materials, reagents, materials, patients, laboratory samples, animals, instrumentation, computing resources, or other analysis tools.
Data Curation	Management activities to annotate (produce metadata), scrub data and maintain research data (including software code, where it is necessary for interpreting the data itself) for initial use and later reuse.
Writing – Original Draft Preparation	Creation and/or presentation of the published work, specifically writing the initial draft (including substantive translation).
Writing – Review & Editing	Preparation, creation and/or presentation of the published work by those from the original research group, specifically critical review, commentary or revision – including pre- or post-publication stages
Visualization	Preparation, creation and/or presentation of the published work, specifically visualization/data presentation.
Supervision	Oversight and leadership responsibility for the research activity planning and execution, including mentorship external to the core team.
Project Administration	Management and coordination responsibility for the research activity planning and execution.
Funding Acquisition	Acquisition of the financial support for the project leading to this publication.

Conceptualization, A.C.S. and A.E.W.; **Methodology**, A.C.S., A.E.W., and W.X.; **Data Curation**, A.E.W., K.M.B; **Formal Analysis:** A.E.W.; **Writing – Original Draft**, A.E.W.; **Writing – Review & Editing**, A.C.S., A.E.W., D.J.L., W.X., K.M.B., J.L.F., A.F.H., Y.C.C., J.L.B., K.L.S., E.J.B., M.J.B, N.M.H., A.B., R.M.R.; **Funding Acquisition**, A.B., R.M.R.; **Supervision**, A.C.S.; **Project Administration**, A.C.S
